# A novel homozygous deletion in ATP6V0A4 causes distal renal tubular acidosis: A case report

**DOI:** 10.1097/MD.0000000000016504

**Published:** 2019-07-26

**Authors:** Jinna Yuan, Ke Huang, Wei Wu, Li Zhang, Guanping Dong

**Affiliations:** Endocrinology Department, Children's Hospital, Zhejiang University School of Medicine, Hangzhou, China.

**Keywords:** *ATP6V0A4* gene, distal renal tubular acidosis (dRTA), homozygous deletion

## Abstract

**Rationale:**

Autosomal recessive distal renal tubular acidosis (dRTA) is a rare condition, most cases of which are caused by genetic mutations. Several loss-of-function mutations in the ATP6V0A4 gene have been recently reported.

**Patient concerns:**

A 2-month, 24-day-old Chinese girl presenting with vomiting and diarrhea.

**Diagnosis:**

dRTA was established by metabolic acidosis and hypokalemia. Mutational analysis of the *ATP6V0A4* gene revealed a homozygous deletion of exons 13 and 14. The father was found to have a heterozygous loss of both exons, whereas the mother was normal.

**Interventions:**

Patient was treated with potassium citrate.

**Outcomes:**

The patient has shown normal pH and potassium levels.

**Lessons:**

This is the first case of a homozygous deletion in *ATP6V0A4* reported in the literature. Although the initial auditory screening was normal in this case, this patient will nevertheless undergo long-term auditory testing.

## 1 Introduction

Bicarbonate is released into the blood via the basolateral chloride–bicarbonate exchanger AE1 (anion exchanger 1, SLC4A1), whereas protons are pumped into urine by vacuolar-type H^+^-ATPases located in the luminal membrane.^[1]^ The kidneys play an important role in the control of acid–base homeostasis. Distal renal tubular acidosis (dRTA) is characterized by impaired urine acidification due to the inability of the distal renal tubule to appropriately excrete H^+^ into the urine.^[2]^ Patients with dRTA develop hyperchloremic metabolic acidosis, usually with a normal anion gap, hypokalemia, failure to thrive, growth retardation, rickets, and nephrolithiasis or nephrocalcinosis.^[3]^ Some patients also present with sensorineural hearing loss (SNHL). Previous studies have shown that most dRTA cases are caused by mutations in the *SLC4A1*, *ATP6V1B1*, and *ATP6V0A4* genes, which encode AE1, transmembrane a4, and catalytic b1 subunits of the apical H^+^-ATPase, respectively.^[4]^ In this study, we report a rare case of dRTA caused by a homozygous deletion of exons 13 and 14 in the *ATP6V0A4* gene.

## 2 Case report

A 2-month, 24-day-old girl was referred to our department presenting with recurrent vomiting. The birth and medical history were uneventful. The patient had metabolic acidosis (pH 7.267, bicarbonate 17.6 mmol/L), alkaline urine (pH 7.5), and hypokalemia (serum potassium 2.4 mmol/L). An ultrasound of the kidneys demonstrated increased echo reflectance at the bilateral medulla. The patient was initially given common treatments to replace fluids and to correct the acidosis and hypokalemia. However, the metabolic acidosis and hypokalemia remained during for 4 days (Table 1). Meanwhile, the hearing, liver function, renal function, count of blood cell, C-reactive protein, and erect abdominal x-ray results were normal. Because hereditary dRTA was suspected, the patient was treated with potassium citrate on day 5 after admission. Since treatment initiation, the patient has shown normal pH and potassium levels.

**Table 1:**
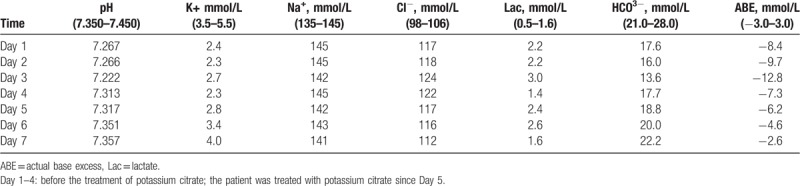
Repeat blood gas analysis during hospitalization before and after the treatment of potassium citrate.

Genomic deoxyribonucleic acid (DNA) was extracted from whole blood using the QIAamp DNA Mini Kit (Qiagen, Shanghai, China) per the manufacturer's instructions. A minimum of 3 μg DNA was used for the indexed Illumina libraries according to the manufacturer's protocol (MyGenostics, Inc., Beijing, China). DNA fragments with sizes ranging from 350 bp to 450 bp and those including the adapter sequences were selected for the DNA libraries. Next, the genes associated with the endocrine system were selected using a gene capture strategy and the GenCap custom enrichment kit (MyGenostics, Inc., Beijing, China) following the manufacturer's protocol. The biotinylated capture probes (80–120-mer), were designed to tile all of the exons with non-repeated regions.

**Figure 1:**
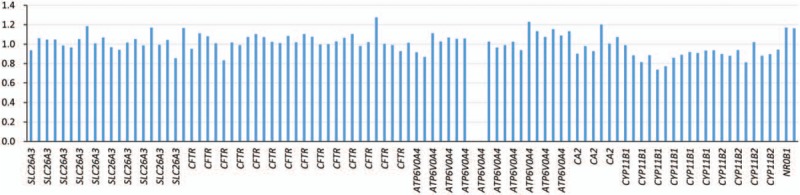
Mutational analysis of the ATP6V0A4 gene revealed homozygous deletion of exon 13 and 14.

**Figure 2:**
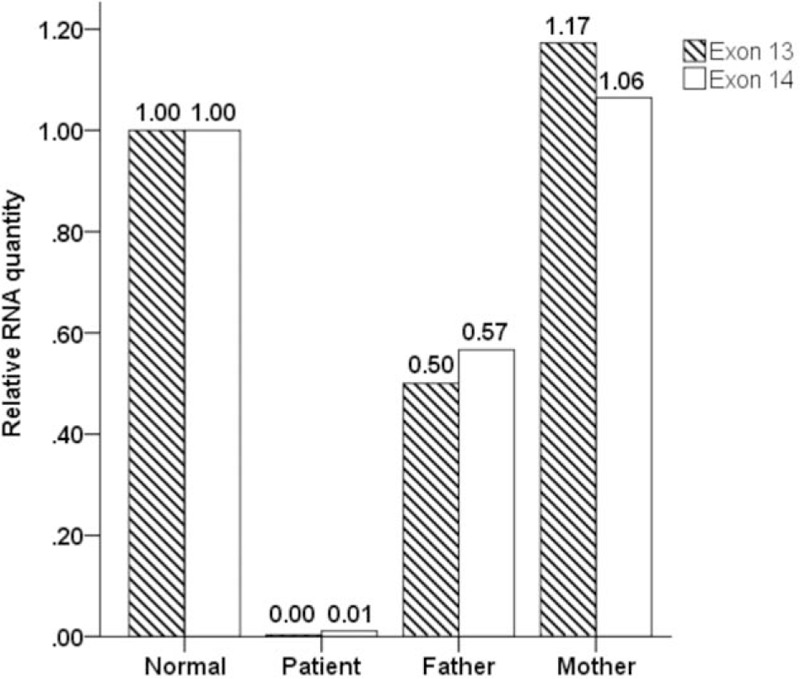
Analysis of exon 13 and 14 of ATP6V0A4 gene by quantitative polymerase chain reaction (PCR). The case is homozygous deletion of exon 13 and 14, the father is heterozygous deletion in the same religions, while the mother is normal.

The patient was found to have a homozygous deletion in exons 13 and 14 of the *ATP6V0A4* gene, which confirmed the diagnosis (Fig. 1). Moreover, quantitative real-time polymerase chain reaction (PCR) (qRT-PCR) of exons 13 and 14 from the *ATP6V0A4* gene using 3 primer pairs was performed on both the patient and her parents using a SYBR@Premix Ex TaqTM (TAKARA, Japan). The Applied Biosystems 7500 real-time PCR system was applied to amplify and quantify the ribonucleic acid (RNA). The relative RNA quantity was calculated based on the comparative Ct and analyzed by the Sequence Detection System software package version 2.0 (PE Applied Biosystems, Carlsbad, CA). The patient's father was found to have a heterozygous deletion of the same regions whereas the mother was found to be normal (Fig. 2).

The parents of the patient have permitted and provided written consent for the publication of this medical data.

## 3 Discussion

Hereditary dRTA is a serious genetic disease that is caused by dysfunction of the alpha-intercalated cells of the cortical collecting duct in the kidney. Both autosomal-dominant (AD) and autosomal-recessive (AR) inheritance patterns have been reported in primary dRTA. *ATP6V0A4* and *ATP6V1B1* mutations are usually associated with AR dRTA, whereas *SLC4A1* mutations are associated with either AD or AR disease. dRTA is a rare disease, and fewer than 30 cases have been reported in China.^[5–13]^ However, >100 genotypes have been reported around the world, and about 10 novel mutations have been reported in Chinese patients.^[11]^Table 2 shows that the most common genotypes are point mutations, whereas deletion mutations are rare in Chinese patients. In this case, the patient was found to have a homozygous deletion in *ATP6V0A4*, whereas her father had a heterozygous loss of exons 13 and 14 in the *ATP6V0A4* gene, and her mother was normal. To our knowledge, this is the first report to show a homozygous deletion of exons 13 and 14 in the *ATP6V0A4* gene.

**Table 2:**
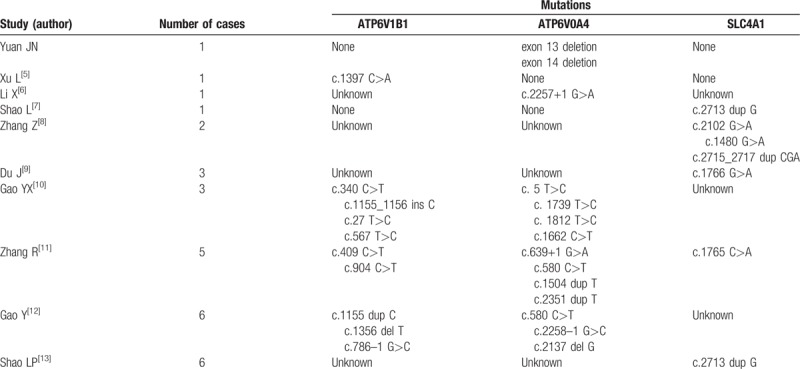
Genotypes of patients with distal renal tubular acidosis reported in China.

The common characteristics of dRTA are hyperchloremic metabolic acidosis accompanied by hypokalemia and relatively normal renal function. In recent years, it was reported that some manifestations of this disease were correlated with the specific genotype (*SLC4A1*, *ATP6V1B1*, or *ATP6V0A4* mutations).^[14]^ Patients with dRTA usually suffer from SNHL,^[15,16]^ but the correlation between the mutation and the development of SNHL remains unknown. One large cohort study showed that the frequencies of SNHL in patients with *ATP6V1B1* and *ATP6V0A4* mutations were 92% and 56.7%, respectively.^[17]^ However, only one patient with a homozygous SLC4A1 mutation was diagnosed with SNHL in that study.^[17]^ Although SNHL has an earlier clinical onset in patients with ATP6V1B1 mutations, it cannot discriminate between *ATP6V1B1* and *ATP6V0A4* mutations.^[18]^ One study in China reported that *ATP6V0A4* mutations were associated with atypical progressive SNHL.^[6]^ Although the SNHL was not found in this patient, auditory evaluations will be performed regularly at follow-up appointments.

Persistent metabolic acidosis of dRTA is associated with osteoporosis and growth retardation. One study reported that stunted growth may be due to the loss of bone minerals and the inadequate production of 1,25 dihydroxycholecalciferol.^[19]^ Furthermore, another study showed that the mechanism of growth retardation in acidosis may be related to a dysfunction of the growth hormone (GH)/insulin-like growth factor (IGF) axis.^[20]^ Fortunately, bicarbonate therapy improves short stature in children with dRTA.^[21]^ In this case, the acidosis was corrected after taking potassium citrate, but we will continue to monitor her growth.

In conclusion, this is the first report to show a homozygous deletion of exons 13 and 14 in the *ATP6V0A4* gene. Although the patient responded well to treatment, auditory and growth evaluations will be regularly performed during the patient's follow-up visits.

## Author contributions

**Investigation:** Jinna Yuan.

**Methodology:** Jinna Yuan.

**Resources:** Guanping Dong.

**Supervision:** Guanping Dong.

**Writing – original draft:** Jinna Yuan.

**Writing – review & editing:** Jinna Yuan, Ke Huang, Wei Wu, Li Zhang, Guanping Dong.
